# Addressing Sexual Dysfunction in Patients with Inflammatory Bowel Disease—We Can Do Better

**DOI:** 10.1093/crocol/otaa007

**Published:** 2020-02-13

**Authors:** Kofi Clarke

**Affiliations:** Division of Gastroenterology and Hepatology, Department of Medicine, Penn State College of Medicine, Hershey, Pennsylvania, USA

Patients with inflammatory bowel disease (IBD) have an increased risk of sexual dysfunction (SD).^1^ The scope of this association is highlighted by the recent study by Riviere et al.^2^ which compared SD in patients with IBD with healthy controls (HC) and patients with irritable bowel syndrome (IBS). Their results show SD rates of 53.6% in IBD versus 28% in HC (*P* < 0.01) and 77.5% in patients with IBS (*P* = 0.10) in women; and 16.9% in IBD, 7.4% in HC (*P* = 0.64), and 26.4% in IBS (*P* = 0.60) in men.

Several previous studies have explored predisposing factors for this association which has a significant negative impact on the quality of life in patients with IBD. These include age and co-morbidity including diabetes, hypertension, previous surgery, biologic use, and severity and activity of the disease. In addition, anxiety and negative body image perceptions including a relative feeling of lack of attractiveness and feeling alone are commonly seen in patients with IBD.^2,3^

Despite this well-described association, a validated, unifying, easy-to-use tool in the office setting that addresses sexual health, disease activity, and severity in patients with IBD is lacking. Separate validated tools including the International Index of Erectile Function have been used in previous SD studies in male patients with IBD.^4^ The National Institutes of Health Patient-Reported Outcomes Measurement Information System has a sexual function and satisfaction measures profile, which has also been used more recently in a study of sexual satisfaction in male patients with IBD.^5^

In this issue of *Crohn’s & Colitis 360*, Gaidos et al. report the findings of a prospective, multicenter Veteran’s affairs study highlighting the high prevalence of SD in male Veterans with IBD: “High Prevalence of Male Sexual Dysfunction in a Prospective Multicenter VA Inflammatory Bowel Disease Population.”

Their results also highlight the underdiagnosis and undertreatment of SD in this cohort. The findings may not be entirely new but add to the growing recognition of a significant problem in the care of patients with IBD.

Incorporating all aspects of comprehensive care including assessing SD into a 30-minute IBD office visit is a daunting challenge. Are providers and patients discouraged by the potential of completing multiple forms or the prospect of an uncomfortable conversation? The development of validated sexual dysfunction scales specific to male and female patients with IBD is a giant step in the right direction.^6,7^

Potential next steps in this line of IBD research should include the development of a validated, *simple to use office-based single tool* combining all pertinent aspects of quality of life including SD in patients with IBD.
